# Integrating Novel Data Streams to Support Biosurveillance in Commercial Livestock Production Systems in Developed Countries: Challenges and Opportunities

**DOI:** 10.3389/fpubh.2015.00074

**Published:** 2015-04-28

**Authors:** M. Carolyn Gates, Lindsey K. Holmstrom, Keith E. Biggers, Tammy R. Beckham

**Affiliations:** ^1^Institute for Infectious Animal Diseases, Texas A&M University, College Station, TX, USA; ^2^EpiCenter, Institute for Veterinary, Animal and Biomedical Sciences, Massey University, Palmerston North, New Zealand; ^3^Texas Center for Applied Technology, Texas A&M University, College Station, TX, USA

**Keywords:** biosurveillance, syndromic surveillance, veterinary medicine, livestock production, infectious disease, information technology, epidemiology

## Abstract

Reducing the burden of emerging and endemic infectious diseases on commercial livestock production systems will require the development of innovative technology platforms that enable information from diverse animal health resources to be collected, analyzed, and communicated in near real-time. In this paper, we review recent initiatives to leverage data routinely observed by farmers, production managers, veterinary practitioners, diagnostic laboratories, regulatory officials, and slaughterhouse inspectors for disease surveillance purposes. The most commonly identified challenges were (1) the lack of standardized systems for recording essential data elements within and between surveillance data streams, (2) the additional time required to collect data elements that are not routinely recorded by participants, (3) the concern over the sharing and use of business sensitive information with regulatory authorities and other data analysts, (4) the difficulty in developing sustainable incentives to maintain long-term program participation, and (5) the limitations in current methods for analyzing and reporting animal health information in a manner that facilitates actionable response. With the significant recent advances in information science, there are many opportunities to develop more sophisticated systems that meet national disease surveillance objectives, while still providing participants with valuable tools and feedback to manage routine animal health concerns.

## Introduction

The recent outbreaks of porcine epidemic diarrhea virus (PEDV) in the United States swine industry (1) and Schmallenberg virus in the European cattle and sheep industries (2) highlight the increasing vulnerability of commercial livestock production systems to emerging infectious diseases. Both outbreaks initially started with animals in a small number of isolated herds displaying unusual clinical signs of severe watery diarrhea and high mortality among suckling pigs for PEDV and fever with reductions in milk yields followed later by the birth of animals with severe congenital defects for Schmallenberg virus. However, by the time, the outbreaks were recognized and confirmed through laboratory diagnostic testing, the viruses had already spread widely across their respective continents due to the high volume of direct and indirect contacts between livestock herds. Conservative estimates of the annual losses from PEDV range from USD $900 million to $1.8 billion depending on the level of piglet mortality assumed by the economic models (3). Although less is known about the cumulative financial impact of Schmallenberg virus in Europe, the average cost of treating individual cases has been estimated at USD $80–$140 per animal (4). Reducing the burden of these diseases as well as future emerging disease outbreaks will require the development of more effective surveillance systems to minimize the delays between disease introduction, detection, and response.

Current methods for detecting emerging infectious diseases in commercial livestock production systems rely heavily on individual veterinarians observing cases with overt clinical signs, pathognomonic lesions, or atypical presentations in the field and then notifying regulatory officials if there is reason to suspect an outbreak, and/or laboratory confirmation of disease and then notifying regulatory officials (5, 6). This can lead to significant delays in detection if farmers decide not to seek veterinary consultation for sick animals, if the clinical signs mimic those of other common endemic diseases, or if the initial cases are observed by different veterinarians who may not be aware that other practitioners are seeing cases with related presentations. Consequently, there has been growing interest in developing biosurveillance systems to integrate pre-diagnostic animal health data from different sources in the livestock industry in real-time so that they can be monitored for unusual spatial or temporal trends that may indicate the presence of an emerging disease concern (6). While these so-called “syndromic” surveillance systems cannot definitively confirm an emerging disease outbreak, they can signal a sufficient probability of an outbreak and alert regulatory officials to clusters of cases that require further epidemiological investigation (7). Feedback from these systems can also be used to enhance the situational awareness of farmers and veterinarians to disease trends in their local region, which may increase the likelihood of voluntarily reporting suspect cases.

**Figure 1:**
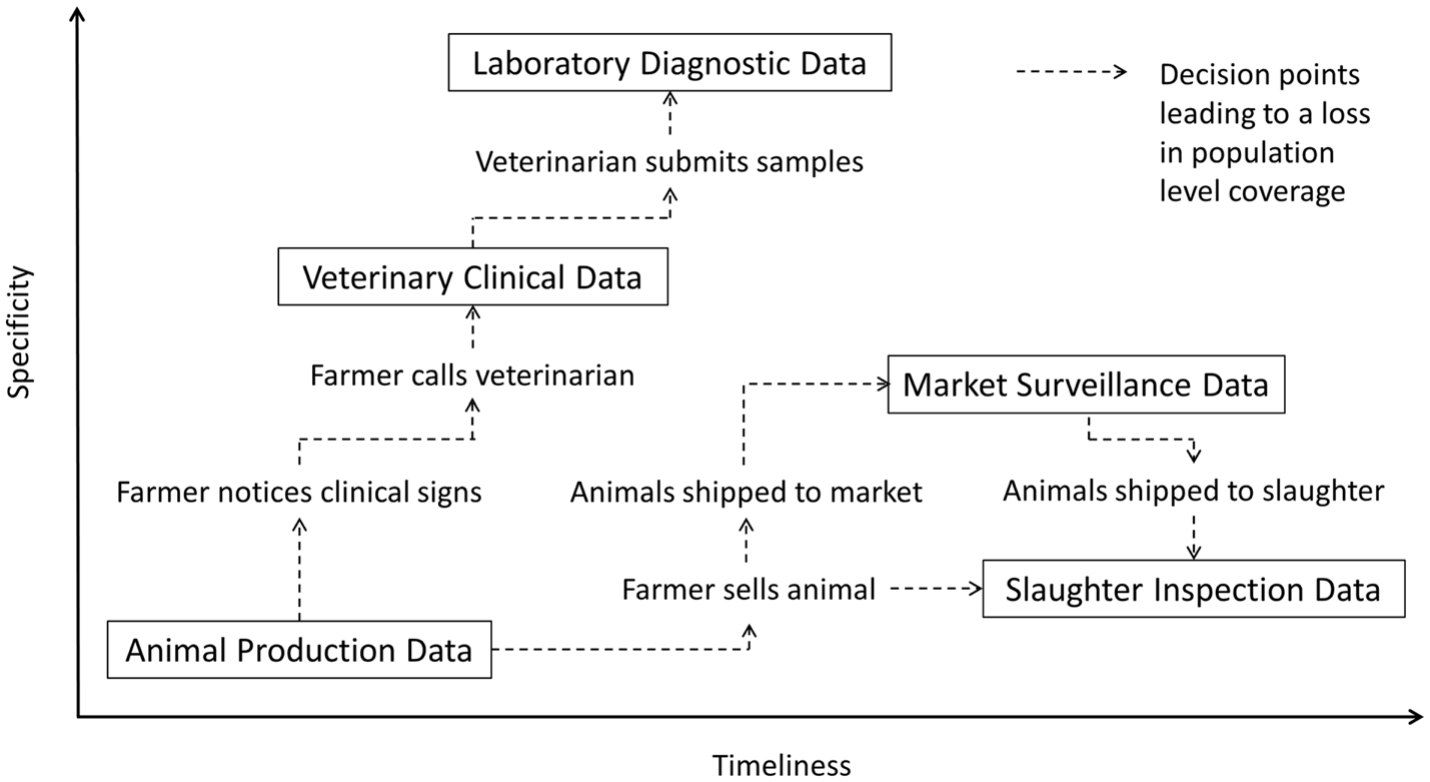
**Relative specificity and timeliness of surveillance data streams in commercial livestock production systems**.

In commercial livestock production systems, the earliest indication that an infectious disease may have been introduced to a farm is often changes in animal health parameters such as feed intake, water intake, activity levels, production levels, reproductive performance, and mortality. This in combination with presence of clinical signs may prompt the farmer to seek advice from a veterinarian. During the farm visit, the veterinarian examines sick animals in the herd, generates a list of differential diagnoses, and then decides whether or not to submit samples for laboratory diagnostic testing. Positive test results may confirm the presence of a known infectious disease agent, while negative test results may indicate the presence of a novel or emerging pathogen. Over the course of this timeline, animals may be shipped to slaughter facilities where the carcasses are examined for lesions as part of routine food safety inspections. Animals that are sold to other farms or slaughter facilities through livestock markets may also be observed by regulatory officials for overt clinical signs of infectious disease as well as may be subjected to further disease-specific diagnostic laboratory testing as part of established national disease surveillance programs. Data collected at any point in this continuum can theoretically be monitored using automated outbreak detection algorithms. However, as highlighted in Figure 1, there are significant differences in the relative specificity, timeliness, and population coverage of each data stream that must be considered when evaluating their use in surveillance systems.

This paper reviews the five primary data streams (animal production data, veterinary clinical data, laboratory diagnostic data, market surveillance data, and slaughter inspection data) that can be used to support infectious disease surveillance in commercial livestock production systems. Particular emphasis is placed on factors influencing data quality and coverage, methods to facilitate data collection in real-time, and insights from published emerging infectious disease surveillance initiatives. The challenges associated with data collection, standardization, analysis, and dissemination are also discussed along with opportunities to improve biosurveillance systems through innovative technology frameworks.

## Data Streams

### Animal production data

Farmers and/or production managers observe animals in their herds or flocks for evidence of disease on a regular basis as part of providing routine husbandry care. The frequency of these observations can depend on many factors, such as the differences in management practices between commercial livestock species (e.g., multiple observations a day on dairy operations, daily observations in poultry and swine operations, less frequent and seasonally dependent for beef cattle kept on pasture). Disease may initially manifest itself as reduced feed and water intake, decreased growth rates, decreased production levels, increased mortality rates, poor fertility, or abnormal behavior well before the appearance of overt clinical signs. With the intensification of commercial livestock production systems, there have been significant advances in developing automated systems for collecting production data to compensate for the decreased time spent observing individual animals in large herds or flocks (8). For example, audio sensors have been installed in swine production units (9, 10) and cattle farms (11) to successfully capture coughing noises and to distinguish those caused by respiratory illness from those caused by poor environmental conditions. In poultry production systems, audio sensor technology has also been used to monitor the feeding behavior of broilers by the intensity and frequency of pecking sounds in the house (12). Other examples in the scientific literature include the accelerometers fitted to halters or collars of dairy cattle to measure jaw movements as an indication of resting, eating, and ruminating periods (13), passive transponder (RFID) tags attached to pigs (14) and cattle (15, 16) to monitor feed intake at controlled feeders, sensors that attaches to teat cup to measure electrical connectivity, color, and milk yield as early warning for mastitis (17) as well as other clinical disorders (18), radiotelemetry units implanted subcutaneously in poultry to monitor heart rate and body temperature (19), and electronic water flow meters used to detect outbreaks of diarrhea in swine (20). In addition, data on the day-to-day inventories, movements, and statuses of herds are routinely recorded to support the business processes of commercial livestock production systems. These data typically reside in databases located within individual operations themselves or within databases of third-party data management companies.

Given the low-economic value of most commercial livestock on an individual animal basis, farmers will often attempt to treat simple conditions identified through direct observation of sick animals or through alerts generated by automated monitoring systems themselves before calling the veterinarian for a farm visit (21). The systems for recording this type of animal health data vary greatly between individual farms with some maintaining highly detailed records of all management and health related events for individual animals using commercial production management software and others keeping only simple paper-based records of treatments for sick animals as required by law in most industrialized countries. Frei et al. (22) conducted a longitudinal study of Swiss dairy herds to evaluate the potential for implementing an intensive animal health data recording system. Over a 15-month period, farmers were required to complete paper-based data sheets for every observed animal health event including information on the date, animal identification, event type, whether or not the veterinarian was called, treatment given, costs, and whether or not laboratory samples were submitted. A list of codes was provided to each farmer to standardize data entry. The average time requirement was approximately 15 min/week and the majority of farmers expressed a willingness to participate in future studies if similar financial compensation was provided.

A more recent study by Menéndez et al. (23) compared the animal health records maintained by Swiss dairy farmers to the records maintained by the farm veterinarian to evaluate the quality of farm-based animal health data. Farmers had the choice of recording data on paper-based forms, electronic spreadsheets, or Internet-based journals and similar data to the Frei et al. (22) study was collected with the addition of information on the name, dosage, and withdrawal time of medications used. There was no difference in the completeness of forms between collection methods with the exception of animal identification being collected less frequently on paper-based forms. Data were missing for approximately 3–7% of the remaining data fields. Farmers recorded significantly more health events than veterinarians (78% compared to 64%); however, the level of agreement (defined as having the same date, event category, and event subcategory) was only 33% on average. The author concluded that it was important to combine farmer data with veterinary data to improve the completeness and accuracy. Other studies have also shown farmers more accurately record data when the events are associated with high treatment costs or significant production losses (24, 25) or during disease outbreaks like the Bluetongue virus epidemic in France (26).

Beltrán-Alcrudo et al. (27) explored the potential for using daily mortality and egg production rates collected from 27 commercial layer flocks in southern California to detect outbreaks of low pathogenic avian influenza (LPAI) faster than through the direct observation of clinical signs, which can be mild or inapparent for many viral strains. Each of the study flocks experienced a confirmed outbreak of LPAI H6N2 during the months of January and February in 2002. Data from 44 other healthy commercial flocks were used to estimate the expected baseline mortality and egg production levels over the production life cycle of a typical commercial layer flock. Alerts were generated when the observed rates exceeded the expected rates by a factor of “*x*” for a single day or by a factor of “*y*” for two consecutive days based on values determined by a previous study in the Netherlands (28). Using low threshold values, the system was capable of detecting all observed outbreaks within 7 days of introduction at the expense of increased false positive signals. Monitoring mortality rates was found to be timelier than monitoring egg production data. However, the authors highlighted the potential for inaccuracies if the mortality data was not collected at a consistent time of day. For example, if mortality data were collected late on 1 day and then at a normal time on the following day, the observed morality rates would be falsely high on the first day and falsely low on the second day. The authors also stressed the importance of using historical data from each flock to calculate expected mortality rates to prevent flocks with chronic management and disease concerns from generating false alerts.

### Veterinary clinical data

Veterinarians visit commercial livestock farms to deliver routine care such as parasite control, reproductive services, regulatory activities (e.g., issue government health certificates), and vaccinations as well as to diagnose and treat animals with clinical illnesses. The frequency of routine visits varies greatly between livestock operations with larger herds and herds using intensive management practices utilizing veterinary services more frequently due in part to the lower average cost of care per animal per visit (29). In addition, commercial livestock operations may also employ a veterinarian(s) within their company. Collecting data from routine visits are important in syndromic surveillance systems to establish that livestock herds are actively being monitored, which can support claims of freedom from disease and be used to calculate denominators in surveillance algorithms (30). The decision to call a veterinarian for clinical illnesses is more complex and based on factors such as the number and economic value of animals affected, previous experience in treating disease, the severity and duration of the clinical signs, and the availability of veterinary services (21, 31–34). This can lead to substantial data loss in veterinary practitioner-based syndromic surveillance systems as well as delays in detecting animals that may be infected with an emerging disease.

Most regulatory authorities require veterinarians to maintain basic medical records with sufficient detail on the animal identification, history, clinical findings, diagnostics, and treatments so that another veterinarian could easily follow the case. Given the remote nature of food animal veterinary work, the majority of practitioners keep medical records in paper format only (35) and must therefore invest additional time in reporting data for surveillance purposes. This has been highlighted as a significant barrier to maintaining veterinary practitioner-based surveillance systems long-term (6). Although some food animal veterinarians use commercial practice management software to maintain electronic records, the software companies may be unwilling to modify their programs to generate automated surveillance data reports since these programs are typically designed for invoice management and not for the electronic transfer of animal health data (35, 36). Furthermore, uptake of these systems has generally been low due to the reluctance of many farmers and veterinarians to modify their existing routines, poor collaboration between software developers, end-users, and data analysts to develop practical interfaces, and the perceived lack of returns on the financial and time investments (37). There is also lack of interoperability between systems that collect similar data (e.g., health certificates, production management information, and laboratory submission forms). In several European countries, veterinarians are also required to submit reports of all bovine farm visits into a national animal health database either through paper-based forms or electronic submissions (38–40). The requirements of veterinarians to enter similar data into multiple systems leads to a decrease in compliance for providing complete information to these different data streams.

The quality of data submitted by veterinarians is highly variable regardless of whether the surveillance program is voluntary or compulsory. In a retrospective study of data collected from seven veterinary practitioners participating in the Ontario swine veterinary-based surveillance (OSVS) pilot program (41), it was found that veterinarians consistently reported basic visit information (farm code, postal code, visit type, and farm production type) and syndromic information (body systems affected), but were less reliable in reporting information on the production parameters affected, type and efficacy of treatments, diagnostic laboratory submissions, and whether the visit was new or related to an ongoing problem. The discrepancies were partly attributed to veterinarians using different definitions for the variable fields than what was provided in the project documentation. It has also been shown that the way veterinarians interpret clinical signs in patients is also highly variable leading to inconsistencies in data recording (42, 43). When submitting mandatory reports on bovine consultations, it has been shown that the completeness of data fields submitted by veterinarians ranges from 17 to 37% for locomotor disorders (39), 56 to 94% for clinical mastitis (40), and 71 to 88% for metabolic disorders (38). Furthermore, survey data from Sweden has also shown that only 18% of veterinarians only reported the main diagnosis for which the animal received prescribed drugs rather than all diseases present (44). The authors of these studies concluded that the data may therefore not accurately reflect the true incidence of disease in the livestock populations.

Detailed reviews on the design of recent voluntary veterinary practitioner-based surveillance initiatives can be found in other published sources (5, 6, 45). Given the limited timespan of available data, their use as early warning systems for disease has been only minimally evaluated. Amezcua et al. (46) analyzed data from the OSVS project to identify clusters of increased report submission rates by season, year, and geographic location using simple regression models. Compared with laboratory test order data from the same time period, the OSVS project identified a greater number of high-risk periods, which corresponded with disease trends in the province. However, no further investigation was performed to determine whether the cases were epidemiologically linked. The authors noted that veterinary compliance with report submission decreased later in the study period as the outbreaks of porcine circovirus associated disease (PCVAD) and porcine reproductive and respiratory syndrome virus (PRRSV) became more distant. A study by Carpenter et al. (47) using data from a large animal health database in Denmark found that outbreak detection tools could potentially reduce the total number of abortions in dairy cattle by 22.9–0.3% depending on the alarm threshold. However, when the cost of abortions was weighed against the cost of responding to an alarm, there were only a limited number of situations where the surveillance system provided any significant financial benefits to the Danish cattle industry.

### Laboratory diagnostic data

Laboratory diagnostic testing is frequently used in conjunction with clinical examinations to determine the underlying cause of disease problems in livestock herds. Samples may also be submitted routinely to diagnostic laboratories as part of national disease surveillance programs, herd health certification schemes, or pre-purchase/movement testing requirements. This data stream has become popular in syndromic surveillance research since most veterinary diagnostic laboratories maintain electronic laboratory information management systems (LIMS). However, the capability to electronically transfer data [e.g., human level seven (HL-7) messaging, web services, custom developed macros] varies greatly between LIMS systems used by the diagnostic laboratories that serve commercial livestock production systems, with LIMS systems used ranging from systems developed in-house to those developed by commercial vendors. Furthermore, many laboratories do not have the information technology (IT) resources and personnel available with expertise to implement this capability, nor have they been fully incentivized so as to have this capability be a mandatory requirement of LIMS systems used. As such, the overwhelmingly majority of laboratory diagnostic data being shared between laboratories and regulatory authorities is done via email and spreadsheets. Those LIMS systems that can be accessed remotely through secure connections to obtain animal health data in near real-time for further analysis most readily support the objectives of syndromic surveillance programs (48).

Veterinarians are required to complete sample submission forms for diagnostic test requests, with the type of information requested on the forms including the date, owner identification, animal identification (age, sex, and breed), relevant clinical and treatment history, specimen characteristics, and diagnostic tests requested. However, there are known issues with the quality and completeness of data recorded on these forms, which are currently primarily paper-based and manually entered into LIMS systems. The test requests can be classified into broad syndromic categories based on the clinical signs associated with the pathogen (49) and monitored for trends that may indicate an increase in the incidence of diseases being observed in the field. Clusters of syndromic cases that test negative for common endemic pathogens may be indicative of an emerging disease threat (50).

The population coverage of laboratory submission data can be influenced by practitioner perceptions and experience (51) as well as the financial and epidemiological state of the livestock industry (52). Based on discussion from a focus group of practicing veterinarians, Robinson et al. (53) found that the high costs associated with performing diagnostic tests deterred sample submission, although producers were more willing to submit samples if the veterinarian was unsure of the diagnosis, if the disease was having a significant economic impact, or if the problem was not resolving with empirical treatment. Similar findings have been reported elsewhere (51, 54).

The likelihood of sample submission also appears to increase if the samples are convenient to collect and the farms are located in closer proximity to diagnostic laboratories (55) and if the diagnostic tests are subsidized through national animal health programs (56). Gilbert et al. (21) estimated that the probability of syndromic cases in the United Kingdom generating an entry in the national laboratory surveillance database ranged from 8.5% for neurologic conditions to 25% for enteric diseases. Outbreaks can also potentially be missed if samples are not of appropriate quality or if the appropriate diagnostic tests are not requested or performed (30). Furthermore, loss in population coverage can occur on submissions to private diagnostic laboratories or from diagnostic tests performed in-house if these data sources are not integrated into surveillance programs.

Several research studies have reported using historical data from veterinary diagnostic laboratories to retrospectively identify disease outbreaks in livestock populations. Hyder et al. (57) scanned data on cattle submission to a national diagnostic laboratory in the United Kingdom and found six clusters of cases where a diagnosis was not reached through laboratory testing. The authors reviewed the accompanying data from the clinical history to determine whether the cases were epidemiologically linked. One cluster may have been caused by a local outbreak of Johne’s disease, while the others were believed to be false positive signals due to the lack of a consistent case definition. This highlighted the importance of collecting good case history information that is easily accessible to allow analysts to quickly distinguish false positive signals from those that require further investigation. The authors also noted potential biases in monitoring cases with no diagnosis for evidence of an emerging disease threat stemming from practitioners requesting limited or pathogen-specific diagnostic testing on their cases. O’Sullivan et al. (50) collected test information on swine samples submitted to a regional veterinary diagnostic laboratory in Ontario to determine whether a known emerging outbreak of PCVAD could be detected by monitoring the weekly proportion of PRRSV tests (an endemic disease with similar clinical characteristics) that returned negative results. A significant association was found for PRRSV PCR results, but not for PRRSV ELISA results, which was attributed to the greater use of PRRSV ELISA for routine monitoring of herd health status rather than for diagnostic purposes during a suspected disease outbreak.

### Market surveillance data

Livestock farmers routinely sell animals to maximize the returns on their available farm resources. This includes selling animals that have reached an appropriate market weight for slaughter, animals that are transferred to other livestock operations for further finishing or as breeding replacements, and animals that have been culled from the herd due to disease, poor performance, or surplus stock. Many countries require animals to be examined by an accredited veterinarian prior to shipment to verify that they are free from notifiable infectious diseases. The corresponding certificates of veterinary inspection may contain information on the shipper, receiver, livestock transport company, date of examination, date and purpose of the movement, animal identification (ear tag or tattoo number, species, age, sex, and breed), animal or herd disease status, and any relevant diagnostic testing results. A study by Portacci et al. (58) evaluated the completeness and legibility of paper-based certificates of veterinary inspection used to accompany cattle shipments within the United States. The authors found that date examination were only recorded on 40% of certificates and many certificates were also missing information on animal identification, which inherently limits the use of this data stream for disease surveillance and livestock traceability purposes. However, there are now options for veterinarians to submit electronic certificates of veterinary inspection using web-based (59–61) and mobile technology platforms (62) to allow for real-time data exchange and improve data legibility and accuracy.

In commercial poultry and swine production systems, farmers often have fixed contracts with other producers and slaughter facilities to transport animals directly between locations when they reach a specified age, weight, and/or production stage. The commercial beef and dairy industries are much less vertically integrated and livestock markets play a more important role in facilitating animal trade. In the United Kingdom, approximately 30% of cattle moved off agricultural holdings pass through livestock markets (63) with a range of statistics reported in countries elsewhere (64–67). Movements of animals through livestock markets are believed to have greatly amplified the spread of foot-and-mouth disease the United Kingdom during the 2001 epidemic (68, 69) and unsurprisingly, there has been interest in developing both active and passive surveillance systems at markets to detect emerging diseases before they become widely distributed.

Van Metre et al. (70) piloted a syndromic surveillance system at a livestock market in the United States, which was based on a trained observer performing visual inspections of animals in each holding pen. Using a paper-based form, data were recorded on the date, the total number of animals in each pen, and the number of animals in the pen showing any of the 12 pre-defined clinical syndromes. Due to privacy concerns, the authors were unable to collect information on the ownership, destination, and demographic characteristics of the animals. Data collection required approximately 2–4 h depending on the volume of livestock entering the market on a given day. Key challenges identified with the system included the difficulty in detecting subtle clinical signs through distant visual observation, inter-observer variability in how animals with clinical signs are categorized into broad syndromic groups, and variation in the production type and demographic characteristics of animals sold on different market days. In a much earlier study estimating the incidence of disease in cattle and swine observed through a livestock market in Saskatchewan, animals with evidence of disease on initial inspection were withheld for a more thorough physical examination to better assess the clinical presentation (71). This may not be feasible at markets with a high volume of livestock trade.

### Slaughter inspection data

In most commercial livestock production systems, animals intended for consumption are subject to ante-mortem and post-mortem examination at slaughter facilities to identify diseases that may pose a risk to human health. The ante-mortem examination involves inspecting animals for abnormal respiration, behavior, gait, posture, discharge, swelling, and other external lesions that warrant segregated slaughter. Data loss may occur since animals with overt clinical signs of disease are not supposed to be transported to slaughter facilities. In the United Kingdom, it has been estimated that 18% of recorded cattle deaths occur on locations other than slaughter facilities (63). After slaughter, the initial internal and external examinations are typically performed by trained meat inspectors on the slaughter line and any carcasses with suspect lesions are withheld for further examination by a federal veterinary inspector to determine whether the product is fit for human consumption. Slaughter facilities maintain basic records of the number and origin of carcasses that are fully or partially condemned for the main purpose of calculating penalties against the submitting producers. The reasons for carcass condemnation are also frequently recorded under broad syndromic categories such as pneumonia, arthritis, emaciation, and abscessation (72). When there is reason to suspect a notifiable disease, samples may be submitted to veterinary diagnostic laboratories for pathogen-specific testing (73).

Several studies have highlighted that the rates of carcass condemnation at slaughter facilities can vary based on other non-biological and non-outbreak factors. For example, Alton et al. (74) found that condemnation rates in provincially inspected abattoirs in Ontario, Canada declined when sales prices were above average, which may be attributed to differences in the quality of animals shipped to slaughter. Higher condemnation rates were also found in abattoirs that accepted a larger proportion of older or poorer quality cattle. The authors concluded that it was important to account for animal age and production class when determining the baseline condemnation rates at slaughter facilities for use in automated surveillance algorithms. Thomas-Bachli et al. (75) evaluated factors contributing to lung and kidney condemnation rates in Ontario swine slaughter facilities. There was significant association between the number of hogs processed by slaughter facilities and lower condemnation rates, which may be explained by the effects of processing speeds on the ability of meat inspectors to identify lesions (76) as well as the possibility that larger slaughter facilities receive higher quality hogs. Seasonality has also been found to influence carcass condemnation rates likely due to changes in management and environmental conditions that change the baseline incidence of disease in livestock populations (77, 78).

Syndromic surveillance systems based on monitoring trends in condemnation rates have successfully been used to detect emerging spatio-temporal clusters in disease incidence. In evaluating historical data from Ontario swine slaughter facilities, Thomas-Bachli et al. (79) identified clusters of high condemnation rates in three slaughter facilities that coincided with known outbreaks of PCVAD, PRRSV, and swine influenza virus (SIV) occurring in the province. Due to privacy constraints and the limitations of analyzing retrospective data, the authors were unable to confirm whether the outbreak signal was caused by animals shipped to slaughter from affected farms. However, in comparison with traditional diagnostic data collected by provincial laboratories in the same study time period, the authors suggested that the slaughter surveillance would have provided an earlier warning of the impending outbreaks. Similar findings were reported in a smaller scale study of data from a single federally inspected slaughter facility in Ontario during the reported outbreaks (80).

## Challenges

### Data collection

For syndromic surveillance systems to be useful in providing an early warning of emerging disease outbreaks, data must be collected and analyzed in near real-time. This can prove challenging given that the majority of farmers, veterinarians, laboratories, markets, and slaughter facilities still rely on paper-based recording systems to manually capture animal health data in the field. These data must subsequently be transferred into electronic databases, which can lead to significant delays before the data become centrally available for analysis. For example, in the Ontario Farm call Surveillance Project (OFSP), the average time from farm visit to report submission was 16 days for paper-based submission forms, 13 days for web-based submission forms, and 7 days for submissions through handheld mobile devices with the majority of participating veterinarians (72 out of 98) choosing to use paper-based submission forms (36). The OSVS pilot program reported that the average time to availability of clinical records was approximately 22 days for both the paper-based submission forms and submissions through handheld mobile devices (41). Furthermore, form completeness is less likely when using paper-based recording systems. For example, when researchers in Sweden compared data from farm copies of veterinary consultation reports against the information to the national animal health database (44), it was found that only 76% of records submitted manually through paper-based forms were complete compared to 95% of records submitted electronically. The discrepancy was largely attributed to the presence of incorrect or unreadable information as well as missing data fields, which are common occurrences when using paper-based recording systems. Options to implement electronic reporting over paper-based forms would help improve the efficiency, completeness, and standardization of data collection and timeliness of data availability for analysis.

### Data security and sharing

Much of the data collected within commercial livestock production systems is considered business sensitive by farmers and veterinarians. In countries without national herd and animal identification programs, there is often reluctance to share identifying farm information with regulatory authorities due to concerns over how the data will be shared and its potential to negatively impact business interests (51, 70). A critical component to the overall success of biosurveillance systems is maintaining the trust of the data providers. Protecting the confidential nature of the data and ensuring that only authorized individuals are provided access to it is essential. Establishing end-user agreements [e.g., memoranda of understanding (MOUs), data sharing agreements] that outline policies for data access, protection, use, sharing, and dissemination can help ensure transparency and enforcement these policies and maintain data confidentiality. An example of this is provided by the United States Centers for Disease Control and Prevention (CDC), which establishes Data Use Agreements with state and local health authorities for data sharing and to conduct syndromic surveillance during peacetime and during health emergencies as part of their BioSense system for public health surveillance (81). In addition, protection of the data within the technology is equally important. Farmers and veterinarians usually prefer their data be housed by a third-party with controlled access to regulatory authorities for surveillance purposes. Having the appropriate mechanisms in place to protect against unauthorized access or accidental release of the data, and to provide access control is necessary. The confidentiality of the data collected must be protected, integrity of the system must be maintained, and system disruptions must be minimized.

### Data standardization

The lack of standardized systems for recording animal health events has been highlighted as a significant barrier to integrating data across multiple data streams and systems (6). Almost every reported syndromic surveillance initiative has collected a different set of variables or used different definitions for the same set or variables. For example, the rapid syndrome validation project for animals (RSVP-A) that collected data from veterinary practitioners in the United States used only 6 syndrome categories (82), whereas a poultry slaughter surveillance program in Brazil recorded 23 common causes for carcass condemnation (78) and a laboratory-based initiative in Canada identified 16 primary syndromic groups based on clinical signs, non-specific diagnoses, or organ systems (49). Previous studies have also found considerable variability in the way different veterinarians interpret clinical cases (42), which may lead to inconsistencies in the types of animal health events that are recorded under each syndrome category. Even in countries with national herd and animal identification programs, it can still be difficult to link animal health databases when the necessary identifying information is not collected appropriately (83, 84). In addition, there is lack of data standardization among diagnostic laboratories, with the naming and coding of the same diagnostic test being highly variable within the LIMS systems among individual laboratories. This makes it difficult for analysts to link information from veterinarians, diagnostic laboratories, markets, and slaughter facilities back to the original farm, prevents the comparability of similar data collected by different systems or within different data streams, and interferes with the calculation of baseline disease incidence rates in outbreak detection algorithms. The sensitivity of the surveillance system for detecting spatial clusters of disease can also be improved when higher granularity data is available for report locations (85–87). Broader usability of the data collected will be enabled by ensuring relevant data fields and categories are standardized or conform to established data standards from other animal health data collection efforts.

### Data analysis

When implemented on a national scale, syndromic surveillance systems are expected to generate large volumes of heterogeneous data that become difficult to analyze using traditional statistical methods. Early changes in disease frequency can easily be masked by the greater natural variation in baseline disease levels observed in large populations (88). The most common solution has been to monitor smaller subsets of data from populations defined by administrative boundaries, geographic locations, or business catchment areas (6). This, however, is also problematic given that livestock disease often spread over wide geographical areas through animal movements (63, 89–91) as was the case during the 2001 foot-and-mouth disease epidemic in the United Kingdom (92). Outbreaks that span across the different monitored data streams may therefore go undetected for longer periods of time. For many outbreak detection algorithms, there are also known issues with accounting for changes in the level of reporting over time due to the recruitment and loss of participants (93), economic and disease factors affecting the industry (52), and changes to the underlying population demographic structure. It has been difficult to evaluate the sensitivity and performance of different analytical approaches in the context of livestock production systems since most published syndromic surveillance projects did not achieve adequate population coverage or were not in operation during known disease outbreaks with sufficient epidemiological data for analysis (6).

### Outbreak response

Most prospective outbreak detection algorithms operate on the same basic principle that when the number of observed cases exceeds the number of expected cases by a specified amount, the system alerts the analyst to a potential emerging disease threat (94, 95). Setting the threshold levels is challenging because of the many uncertainties in how an emerging infectious disease threat will appear as a signal in the syndromic surveillance data. If the threshold levels are set too high, there may be delays in detecting true disease outbreaks, which can lead to larger outbreak sizes and significantly greater socioeconomic impacts. If the threshold levels are set too low, there will be an increased frequency of false positive alerts, which can lead to user fatigue, resource depletion, and decreased confidence in the system’s performance. Furthermore, monitoring multiple data streams simultaneously is also likely to increase the absolute number of false positive alerts generated by the system. A recent review by Rolka et al. (96) highlighted numerous other challenges with monitoring multiple data streams including poor alignment in the coverage and timeliness of different data sources, difficulty in linking data streams to obtain accurate estimates of outbreak size, the theoretical nature of proposed statistical methods for integrating data from multiple sources, and the need for better visual analytics and decision support tools to facilitate rapid outbreak response.

### Program sustainability

With the exception of a few Scandinavian countries, the participation of farmers and veterinarians in syndromic surveillance initiatives has typically been on a voluntary basis. For that reason, many pilot projects have provided incentives such as direct financial compensation (51) or credits toward laboratory diagnostic testing (36) as a means of encouraging participants to submit surveillance reports. Zurbrigg and Van den Borre, (36) demonstrated a significant increase in the timeliness of report submissions in the OFSP during the time period when participating veterinarians were reimbursed for conducting post-mortem examinations on-farm. Follow-up survey studies have also revealed that both farmers (22) and veterinarians (41) believe that financial compensation for the time spent collecting data is essential for long-term project sustainability, although some participants were willing to continue submitting data voluntarily because of the value they perceived in conducting infectious disease surveillance. This raises concerns over the sustainability of large scale syndromic surveillance programs without a continued source of funding or building incentives into the program that benefit farmers and veterinarians so they see value in the system for managing animal health at the farm level. Participants have also expressed frustration that aggregate information on disease trends or significant findings was not made available in a useful format (53) and that the disease situation of livestock populations has not actually improved despite the significant time and resources being invested in surveillance programs (51).

## Discussion

The long-term success of syndromic surveillance programs in commercial livestock production systems hinges on being able to use innovative technology platforms to integrate animal health information from diverse data sources into a common operating picture where it can be used to support emerging infectious disease detection and decision-making as well as efforts to manage endemic diseases more cost-effectively at the farm and industry levels.

A key step toward improving the quality and timeliness of animal health data collected through syndromic surveillance systems will be developing mobile technology platforms that allow participants to capture information electronically as part of their normal work routines (23). The VetPad initiative in New Zealand is one example where veterinarians were provided with an interface for handheld mobile devices that operated as a practice management software as an incentive to submit surveillance reports (97). The main advantage over paper-based recording systems is the ability to standardize data collection by making key data fields required before submission to prevent data loss and by providing pre-determined lists or validation constraints for each data field to ensure consistency in how the information is recorded. Reducing the need for double data entry, such as mobile technology capabilities to allow data collected to be submitted for multiple purposes (e.g., an electronic laboratory submission form being automatically generated from a syndromic surveillance report) and integrating tools to automatically transfer completed reports into a centralized database is also likely to increase long-term compliance by minimizing the time burden on program participants (36). Several commercial herd and veterinary practice management software programs also offer users the option of recording data through interfaces designed to operate on personal digital assistants (PDAs), smartphones, or tablets in the field. However, variability in the type and format of recorded data makes it difficult to integrate into syndromic surveillance initiatives without either making significant modifications to the underlying source code or creating independent software programs that map local terminology into a standardized coding system. This can be addressed using innovative technology solutions that allow for the interoperability, and therefore data integration between different IT systems. This challenge highlights the need to establish national and international standards for reporting animal health information consistently at all stages in the production chain, which includes establishing definitions to standardized terminology and ensuring they are properly understood by program participants. This can be achieved through training and by having this information be easily referenced within the surveillance forms. In addition, evaluations of the data collected should be performed to ensure their use fit intended purposes of the syndromic surveillance system (e.g., assess frequency of use and trends, evaluate training, compare syndromic categorizations with diagnostic tests results). Data standardization and consistency are also needed within diagnostic laboratories, including the collection, naming convention, and coding of data within LIMS systems. A working group of epidemiologists in Canada recently published a list of minimum data requirements to support disease surveillance using diagnostic laboratory submissions (98). These included the (1) unique laboratory submission identifier, (2) unique premises identifier, (3) sample submission date, (4) geographic location for the premises, (5) species tested, (6) main farm type, (7) production type of animals tested, (8) total population of the species tested on the farm, (9) total number sick, (10) total number dead, (11) diagnostic test(s) performed, (12) disease agent(s) screened, (13) test results, (14) syndromic classification, and (15) final diagnosis. Subjective elements such as risk factors (husbandry practices, farm demographics, animal characteristics), clinical information (clinical signs, differential diagnoses, treatments, laboratory submissions), potential confounders (feed changes, facility issues, environmental conditions), and other case notes were excluded from the list on the basis of being time consuming to collect and highly variable in how they are reported by different practitioners. However, this additional information can be used by analysts to determine whether cases in an identified cluster are epidemiologically linked (57) as well as to provide more useful feedback to farmers and veterinarians on disease management (99). As mentioned above, providing mechanisms for practitioners to electronically submit laboratory submission forms (e.g., online submission forms, mobile applications) would help reduce the time burden of completing these forms and allow diagnostic laboratories to require certain data fields be included on all submissions, such as these minimum data requirements. Education and outreach to practitioners on the value and benefit of providing more information on laboratory submission forms to manage the health of their clients’ animals is needed to achieve better compliance, as well as initiatives for diagnostic laboratories themselves play a larger role in syndromic surveillance programs and provide useful information back in a consumable format as a service to their clients.

Another possible solution is to ensure that the different, but complementary information recorded by the various data streams can be linked through either herd or animal identification numbers. Glass-Kaastra et al. (100), for example, used both clinical and laboratory data from the OSVS program to characterize patterns in antimicrobial use and risk factors for treatment failure to help veterinarians select the most appropriate treatments for their patients and to help regulatory officials monitor livestock populations for evidence of antimicrobial resistance. As the use of RFID identification tags on livestock expands, it will also become easier to track production and health parameters on individual animals from birth through slaughter (101). Although some farmers have expressed concerns over sharing identifying information, there are now much more sophisticated technology platforms that can provide relevant summary statistics to key industry stakeholders while still protecting confidentiality. There are several examples in the literature where aggregate slaughter surveillance data from national animal health schemes has been successfully shared with participants for the purpose of benchmarking the performance of their farms against others in the industry (102–104).

The consistent use of herd and animal identification numbers in surveillance reports also facilitates the development of better statistical methods to detect emerging disease outbreaks. In countries where detailed information on livestock movements and farm locations is available through national computerized databases, it may be possible to strategically select space-time windows using network-based approaches to avoid the problem of using artificial boundaries to subset the large volumes of surveillance data. This process first involves reconstructing the contact network by creating links between farms that trade animals or are located in close proximity. These links may be weighted by the volume and frequency of animals traded in the case of movements or by the distance between farms in the case of spatial proximity. Various community detection algorithms can then be used to divide the population of farms into linked networks or communities based on the strength of connections between them (105, 106). Theory holds that if an infectious disease is introduced to a livestock farm, it has a greater chance of spreading to other farms within the community than to farms outside the community. It may also be worth establishing temporary subsets of farms cased on their co-attendance at livestock events as markets, rodeos, or shows, since there is high risk of disease being introduced and widely disseminated through these venues (107, 108).

Several methods have been proposed to reduce the error caused by setting arbitrary threshold values. Dórea et al. (109) developed an approach based on aggregating the results from multiple outbreak detection algorithms that were run simultaneously on laboratory submission count data. Rather than setting a single threshold value, outbreak alert signals were assigned a “severity” score based on how far they deviated from the expected baseline values. The severity scores for the different algorithms were then combined and an alert was generated if the overall score exceeded another preset threshold value. This approach may increase the sensitivity of the system to diseases with a slow increase in case counts. Carpenter et al. (110) suggested using a two-level approach to determine the level of response to outbreak signals from data on abortions in dairy cattle. When the difference between the number of observed cases and the number of expected cases exceeds the threshold value once in a given time period, there should be only a limited preliminary investigation into patient risk factors. If the number of observed cases continues to exceed the number of expected cases in consecutive time periods or if the magnitude of the difference is excessively large, then there should be a more involved field investigation and/or outbreak response. A third approach used by Amezcua et al. (46) in the context of swine disease surveillance was to compare trends observed in the syndromic reports submitted by participating veterinarians to the corresponding laboratory submission count data from the same time period. The observation of similar trends may increase suspicion that the alert signal represents a true disease outbreak.

Some of the basic principles from risk-based surveillance (111) may also be useful in setting threshold values for outbreak detection algorithms. Certain farms are known to have a high risk of acquiring and spreading disease based on their connectivity in the animal network, proximity to other farms, demographic characteristics, and biosecurity practices. These factors could be used to generate a risk score for individual farms. The threshold values required to trigger an alert could then be varied according to the aggregated risk scores for all farms present in the outbreak cluster. The basic premise is to increase the timeliness of response in situations where there is a high risk of disease spreading rapidly from the index farms. Similarly, diseases that present with an unusually high morbidity and mortality or unusually severe clinical signs should trigger an alert at lower thresholds than diseases with mild case presentations. Proper evaluation of these statistical methods will require the development of synthetic datasets to compensate for the lack of data with sufficient population coverage, duration, and superimposed natural disease outbreaks from pilot surveillance projects. In the public health field, there is a growing field focusing on the development of synthetic syndromic surveillance datasets to protect patient confidentiality while still providing researchers realistic enough baseline data to support methodological investigations (112). The veterinary community would benefit from efforts to develop similar synthetic datasets for livestock populations.

With the increasing sophistication of the technology platforms supporting syndromic surveillance efforts, it is also possible to provide participating farmers and veterinarians with customized tools to improve animal health management. This has been identified as an important incentive for continued participation (36). It has also been well established that the use of information management systems can offer significant financial returns through increased productivity (113), which may help farmers see the value in adopting electronic recording systems. In the BOSS project from Australia, which was designed to collect syndromic surveillance data from remote beef cattle herds, researchers developed a Bayesian classification system to provide participating producers with the most likely diagnosis based on the submitted clinical signs (114). A similar system has been proposed to use the clinical signs reported in veterinary practitioner-based surveillance data to identify cases with presentations that are compatible with known transboundary animal diseases such as bluetongue virus (115). Other valuable tools may include the ability to automatically detect herds with a higher incidence of disease or poorer performance than the general population based on established benchmarks, summary reports of disease trends in the surrounding region to increase situational awareness of local disease concerns, and systems that allow farmers and veterinarians to easily track the efficacy of different management interventions by comparing production parameters before and after change. Establishing the use of syndromic surveillance for purposes beyond emerging infectious disease detection is important for justifying the costs of implementation and ensuring its sustainability (116).

## Conclusion

As highlighted by this review, there is still much to be learned about how data collected from farmers, veterinarians, diagnostic laboratories, markets, and slaughter facilities can be used to support infectious disease surveillance in commercial livestock production systems. Each data stream has its own unique challenges associated with achieving adequate specificity, timeliness, and population coverage. However, advances in IT are greatly expanding opportunities to collect and integrate animal health data in real-time for use in detecting emerging infectious disease outbreaks as well as for managing common endemic diseases more cost-effectively than traditional surveillance systems.

## Author Contributions

MG conducted the literature review and drafted the manuscript. TB, KB, and LH provided scientific guidance on the manuscript structure and contents. All authors read and approved the final manuscript.

## Conflict of Interest Statement

The authors declare that the research was conducted in the absence of any commercial or financial relationships that could be construed as a potential conflict of interest.
